# Transcription factor GATA6 promotes migration of human coronary artery smooth muscle cells *in vitro*

**DOI:** 10.3389/fphys.2022.1054819

**Published:** 2022-11-29

**Authors:** Azra Alajbegovic, Fatima Daoud, Neserin Ali, Katarzyna Kawka, Johan Holmberg, Sebastian Albinsson

**Affiliations:** ^1^ Department of Experimental Medical Science, Lund University, Lund, Sweden; ^2^ Department of Physiology and Biochemistry, School of Medicine, The University of Jordan, Amman, Jordan; ^3^ Department of Clinical Sciences Lund, Orthopedics, Clinical Epidemiology Unit, Lund University, Lund, Sweden

**Keywords:** GATA6, vascular smooth muscle, cell migration, microarray, phenotypic modulation

## Abstract

Vascular smooth muscle cell plasticity plays a pivotal role in the pathophysiology of vascular diseases. Despite compelling evidence demonstrating the importance of transcription factor GATA6 in vascular smooth muscle, the functional role of GATA6 remains poorly understood. The aim of this study was to elucidate the role of GATA6 on cell migration and to gain insight into GATA6-sensitive genes in smooth muscle. We found that overexpression of GATA6 promotes migration of human coronary artery smooth muscle cells *in vitro,* and that silencing of GATA6 in smooth muscle cells resulted in reduced cellular motility. Furthermore, a complete microarray screen of GATA6-sensitive gene transcription resulted in 739 upregulated and 248 downregulated genes. Pathways enrichment analysis showed involvement of transforming growth factor beta (TGF-β) signaling which was validated by measuring mRNA expression level of several members. Furthermore, master regulators prediction based on microarray data revealed several members of (mitogen activated protein kinase) MAPK pathway as a master regulators, reflecting involvement of MAPK pathway also. Our findings provide further insights into the functional role of GATA6 in vascular smooth muscle and suggest that targeting GATA6 may constitute as a new approach to inhibit vascular smooth muscle migration.

## Introduction

Vascular diseases are among the leading causes for morbidity and mortality worldwide. A large body of work has recognized that vascular smooth muscle cells (VSMCs) play a prominent role in these pathological processes. Unlike other terminally differentiated cells, VSMCs retain a remarkable capability to undergo phenotypic modulation. In response to changes in the surrounding environment, VSMCs can switch from a differentiated contractile phenotype to a more proliferative and migratory phenotype, often referred to as a synthetic phenotype. Although this is beneficial during various biological processes such as wound repair, phenotypic modulation can play a fundamental role in the development of various vascular diseases, including atherosclerosis, hypertension and restenosis following angioplasty (Owens, 1995; Owens et al., 2004).

SMC migration is a key process in the development of coronary artery disease. SMCs respond to a perceived injury to the vascular wall by migrating from the vascular media towards the lumen where they proliferate and form a neointima. Eventually, this effect together with an atherosclerotic process can limit blood flow and increase the risk of thrombosis. Although synthetic smooth muscle cells often have both increased proliferative and migratory capacity, these are separate biological processes that are regulated by distinct signaling pathways (Wang et al., 2003).

Despite extensive effort to characterize the transcriptional program that defines smooth muscle phenotype, the role of endogenous regulators that control smooth muscle specific gene expression are not fully understood. One of the transcription factors that appears to have a complex role in smooth muscle gene expression is GATA6, which is the predominantly expressed GATA factor in VSMCs (Morrisey et al., 1996; Narita et al., 1996). GATA6 belongs to a family of highly conserved zinc-finger transcription factors that regulates the expression of genes required for developmental processes and tissue-specific functions. Several studies have demonstrated a role for GATA6 in maintaining the differentiated state of VSMCs by regulating the expression of smooth muscle-specific genes including smooth muscle myosin heavy chain (*MYH11*) (Wada et al., 2002) and smooth muscle alpha actin (*ACTA2*) (Kanematsu et al., 2007). Moreover, GATA6 has been shown to reduce smooth muscle proliferation (Perlman et al., 1998) and neointimal formation *in vivo* following balloon injury in mice (Mano et al., 1999; Zhuang et al., 2019). Consistent with these findings, rapamycin, a common stent drug preventing restenosis, mediates positive effects on SMC differentiation and prevents vascular disease by phosphorylation-mediated activation of GATA6 (Xie et al., 2015). However, several studies have demonstrated that GATA6 can promote expression of genes associated with the synthetic function of SMCs(Lepore et al., 2005; Yin and Herring, 2005). This effect of GATA6 may be caused by inhibitory or activating interaction with myocardin, a master regulator of SMC identity, depending on the target gene (Oh et al., 2004; Yin and Herring, 2005). Thus, GATA6 appears to play multifaceted roles in the regulation of smooth muscle phenotype and exploring the molecular action of GATA6 may have pivotal implications for our understanding of smooth muscle cell function and the underlying mechanisms of vascular disease.

The aim of this study was to further elucidate the importance of GATA6 for human vascular smooth muscle gene expression and cell migration. Surprisingly, our results suggest that GATA6 promotes cell migration of human coronary artery smooth muscle cells (HCASMCs). Furthermore, a microarray screen of GATA6-sensitive gene transcription identified several members of the transforming growth factor beta (TGF-β) signaling.

## Material and methods

### Cell culture

Primary human coronary artery smooth muscle cells (HCASMCs) from two different donors (Lot: 1130140 and 1689414) were purchased from Gibco Life Technologies (#C-017-5C) and maintained in Medium 231 (Life Technologies, #M231500) supplemented with 5% smooth muscle growth supplement (Life Technologies, #S-007–25) and 1% penicillin/streptomycin (Biochrom, #A2212). Cells were cultured at 37°C in 5% CO_2_ and used until passage 8. Media was changed every other day.

### Adenovirus transduction

Overexpression of human GATA6 gene was achieved using adenoviral constructs. Cells were transduced 24 h after seeding with 100 MOI of Ad-CMV-GATA6 (Vector Biolabs, #1027). Fresh media was added 96 h after virus transduction for additional 48 h. Ad-CMV-null (Vector Biolabs) was used as a control.

### GapmeR transfection

Cells were transfected with GATA6 or Negative Control GapmeR (Qiagen, 10 nM) for 96 h using Oligofectamine transfection reagent (Life Technologies) according to manufacturer’s instructions. Cells were transfected 24 h after seeding, at approximately 40–50% confluency with GATA6 or Negative Control GapmeR (Qiagen, 10 nM). Transfection was performed in Oligofectamine transfection reagent (Life Technologies) using Opti-MEM reduced-serum medium (Life Technologies, #11058–021) according to manufacturer’s instructions. After 15 h, Medium M231, supplemented with 2× the normal concentration serum and penicillin/streptomycin, was added to the cells without removing the transfection mixture. After an additional 81 h, cells were analyzed for migration and gene expression.

### Cell migration assay

Migration assay was carried out using a scratch wound healing assay and a transwell migration assay. For wound healing, cells were seeded in culture plates and treated as indicated on the following day. After 6 days, scratch wounds were created with a pipette tip. Cells were serum-starved overnight to synchronize the cell cycle and prevent cell proliferation being a factor to affect migration. Images were taken with a phase-contrast microscope (Olympus CKX41 microscope, CellSens Dimension software) at indicated time points. Migration was assessed by analyzing the migrated distance (gap width at indicated time point/gap width at 0 h) using ImageJ software.

For transwell migration assay, cells were seeded in cell culture flasks and treated accordingly. Following treatment with virus or GapmeRs the cells were serum-starved overnight before trypsinization and resuspension in serum-free medium. A total of 250 μL of cell suspension (10^5^ cells/ml) was added to cell culture inserts (Corning, Cell culture inserts with 8.0 µm pores) and 5% smooth muscle growth medium was added in the lower compartment (Corning, 24-well plate) to induce cell migration. After 24 h of incubation, non-migrated cells were removed with cotton tips and migrated cells at the bottom of the inserts were fixed with 1% glutaraldehyde in Hank’s balanced salt solution (HBSS) and nuclear stained with 0.1% Crystal violet. Images were taken with a phase-contrast microscope (Olympus CKX41 microscope, CellSens Dimension software). After that, 10% acetic acid was added to the wells and incubated for 5 min to dissolve crystal violet. Supernatants were transferred to 96 well-plate and absorbance was measured at 595 nm with a spectrophotometer. Each analysis was performed in triplicates.

### Digital holographic cytometry

HoloMonitor M4, a label-free imaging technology from Phase Holographic Imaging (PHI, Lund, Sweden), was used to analyze single cell motility. Cell motility was quantified by recording a time-lapse sequence of migrating cells in standard culture conditions. Briefly, cells were seeded in 6-well culture plates (Sarstedt #83.3920.005) and transfected with GapmeRs as described previously. On day four following transfection a pipette tip was used to create a scratch wound to induce cell migration. The lid was replaced with PHI HoloLids^TM^ imaging covers, sterilized in 70% ethanol. The plate was placed onto a HoloMonitor microscope inside an incubator and set to automatically capture images at 20 min intervals for 36 h using the Hstudio software. For reproducibility, multiple fields and wells were analyzed. The number of cells that migrated to the wound area and their migration speed and distance was obtained by tracking individual cells over time.

### Quantitative RT-PCR

Isolation of total RNA and qPCR were performed as described previously (Turczynska et al., 2015). Expression of mRNA was determined using commercially available primers from QuantiTect Primer assays (Qiagen): Hs_ACTA2_1_SG (#QT00088192), Hs_CNN1_1_SG (# QT00067718), Hs_SYNPO2_1_SG (# QT00075614), Hs_MYH11_1_SG (# QT00069391), Hs_BMP2_1_SG (QT00012544), Hs_BMP4_1_SG (QT00012033), Hs_TGFB3_1_SG (QT00001302), Hs_TGFB2_1_SG (QT00025718), Hs_SMAD1_1_SG (QT00103824), Hs_SMAD3_1_SG (QT00008729), Hs_RRN18S_1_SG (QT00199367). Primers for GATA6 (sense: 5′-GTG​CCT​TCA​TCA​CGG​CGG​CT, antisense: 5′-CAC​ACG​GGT​TCA​CCC​TCG​GC) were purchased from Europhins.

### Western blotting

Cultured cells were lysed in Laemmli buffer (60 mM Tris-HCl, pH 6.8, 2% SDS, 10% glycerol) supplemented with protease (Sigma-Aldrich #P8340) and phosphatase inhibitors (Thermo Fisher #78420). Protein determination was performed using the DC protein assay from Bio-Rad. Bromophenol blue and β-mercaptoethanol were added to samples at final concentrations of 0.02% and 8%, respectively. Equal amount of protein (20 µg) was loaded in each lane on Bio-Rad Criterion TGX 4–15% gels followed by semi-dry transfer to nitrocellulose membranes using the Trans-Blot Turbo system (Bio-Rad). Proteins were detected using commercially available primary antibodies against GATA6 (Cell Signaling #4253S, 1:1,000), α-actin (Sigma-Aldrich #A5228, 1:1,000), smooth muscle myosin heavy chain (abcam #ab53219, 1:1,000), calponin (abcam #ab46794, 1:1,000), SMAD1 (cell signaling, #9743, 1:1000), SMAD3 (cell signaling, SMAD 2/3 antibody, #3102, 1:1000), Hsp90 (BD Biosciences #610418, 1:10,000) at 4°C overnight. HRP-conjugated secondary antibodies anti-mouse (Cell signaling #7076) and anti-rabbit (Cell signaling #7074) were incubated for 1 h at room temperature. Bands were detected by chemiluminescence (SuperSignal West Femto Maximum Sensitivity Substrate, Thermo Fischer). Images were acquired in an Odyssey Fc Imager (LI-COR Biosciences). Several proteins were detected on separate blots from a single TGX gel. HSP90 from each lane was used as loading control for all proteins detected on a single gel in the respective lane (see Supplementary Material).

### Microarray analysis

Human coronary artery smooth muscle cells were transduced 24 h after seeding with 100 MOI of Ad-CMV-GATA6 or Ad-CMV-null. Fresh media was added after 96 h and cells were kept in culture for another 48 h. RNA was extracted using the RNeasy mini kit (Qiagen #74106) according to the manufacturer’s instructions. RNA quality was determined using a 2,100 Bioanalyzer instrument (Agilent). Total RNA was analyzed by Affymetrix GeneChip Human Gene ST Array by the Swegene Center for Integrative Biology at Lund University (SCIBLU). The microarray data is accessible *via* the Gene Expression Omnibus (accession number GSE216686, scheduled release on October 31st 2022).

Pathways analysis of all differentially expressed genes was performed using PANTHER 17.0 version and false discovery rate <0.05 was considered significant. The differentially expressed genes were also analyzed by QIAGEN Ingenuity Pathway Analysis (IPA) software to evaluate common interactions. Differentially expressed genes (242 downregulated and 696 upregulated) were mapped and compared to the whole data set consisting of 19,683 genes as a background data set. The software was unable to map 1,213 genes. Therefore, these genes were not further included in the pathway analysis. A prediction of the master regulators was done by IPA software by comparing changes in our dataset with up-to-date literature. The master regulators were given a Z-scores, a positive Z-score indicates activation status and a negative Z-score indicates inhibition status, taking into account the direction of the changed genes. After sorting the master regulators by their absolute Z-score, the top 100 master regulators were visualized as a network to show the overall interaction between the master regulators using STRING database version 11.5. The color shades represent the degree of activation/inhibition ranging from dark blue (most active) to dark red (most inhibited). The line’s color indicates the strength of data that support such interaction.

### Statistics

Results are presented as mean ± standard error of the mean (S.E.M). Statistical analysis was performed using GraphPad Prism Software 5. Comparisons between two groups were analyzed using the student’s *t*-test. Number of replicates for each experiment is specified in the figure legends. *p*-values less than 0.05 were considered statistically significant.

## Results

### GATA6 promotes expression of smooth muscle marker genes

**FIGURE 1:**
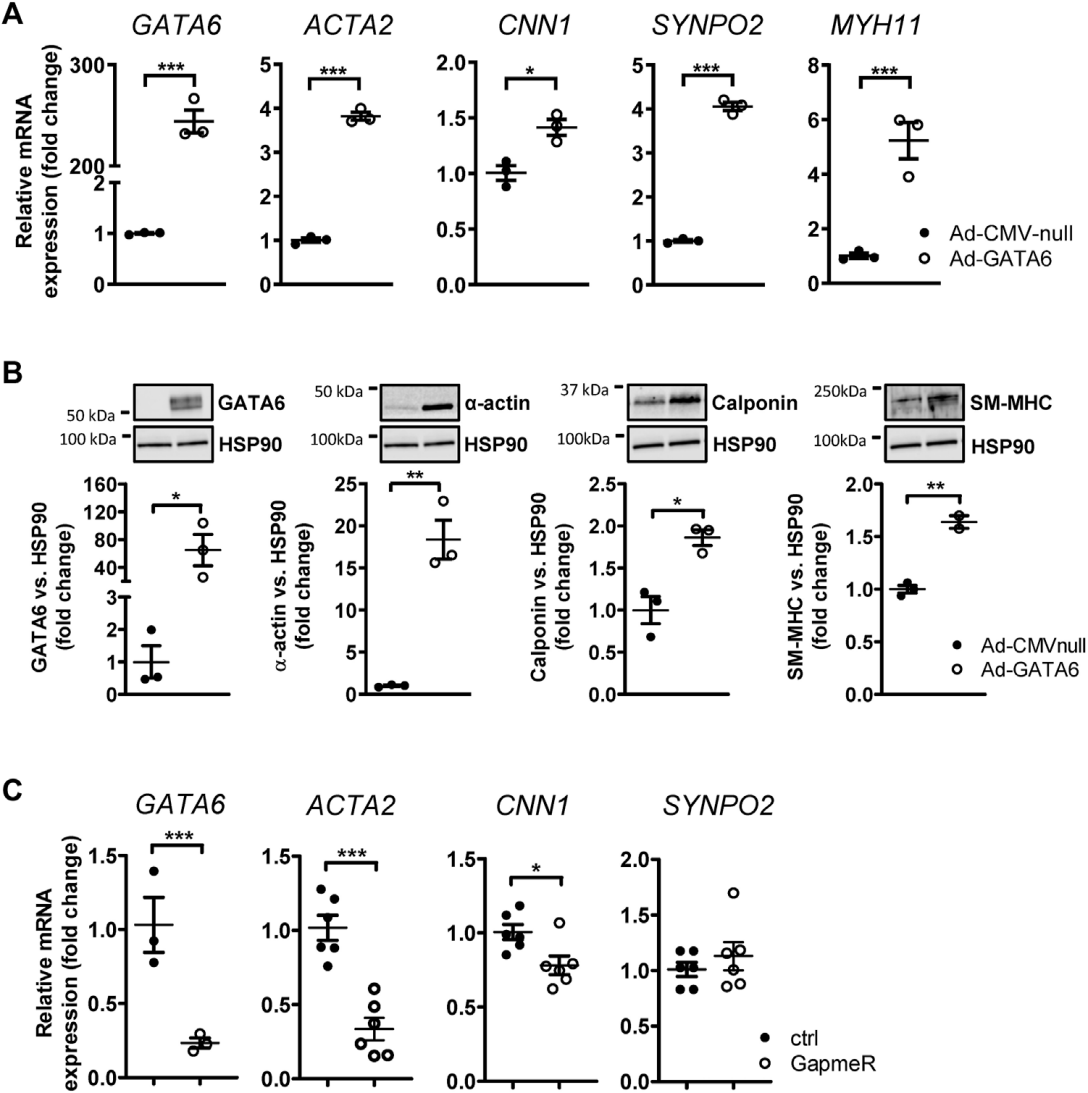
GATA6 induces vascular smooth muscle cell differentiation. Human coronary artery smooth muscle cells were transduced with Ad-CMV-GATA6/Ad-CMV-null **(A,B)**. Expression of GATA6 and some smooth muscle markers are presented in **(A)** at mRNA level and **(B)** at protein level. **(C)** Relative mRNA expression of targets after transfection with GapmeRs against GATA6. Data are presented as mean ± SEM (*n* = 3–6). *p < 0.05, **p < 0.01, ***p < 0.001. α-actin (*ACTA2*), smooth muscle myosin heavy chain (SM-MHC, *MYH11*), calponin (*CNN1*), and synaptopodin-2 (*SYNPO2*).

The differentiation status of smooth muscle can be evaluated by expression of genes involved in the contractile function of SMCs(Owens et al., 2004). Although accumulating studies state the importance of GATA6 in maintaining the differentiated state of SMCs, *in vitro* and *in vivo* data has demonstrated that GATA6 is not necessarily required for the expression of SMC-restricted genes (Lepore et al., 2005; Lepore et al., 2006). To determine the effect of GATA6 on smooth muscle differentiation we analyzed the expression of SMC marker genes following GATA6-induction. HCASMCs were transduced with 100 MOI GATA6 (Ad-CMV-GATA6) or vehicle (Ad-CMV-null) for 6 days. Since expression levels of GATA6 in cultured SMCs is relatively low, transduction with Ad-CMV-GATA 6 resulted in a substantial induction of GATA6 mRNA by ≈ 245 fold and protein level by 64 fold (Figures 1A,B). Our results confirmed previous findings demonstrating a positive effect of GATA6 on VSMC differentiation (Xie et al., 2015). We show that GATA6 stimulates the expression of genes associated with the differentiated phenotype of SMCs including smooth muscle myosin heavy chain (*MYH11*), synaptopodin 2 (*SYNPO2*), calponin (*CNN1*) and smooth muscle alpha-actin (*ACTA2*) at the mRNA and/or protein level (Figures 1A,B). To assess the role of GATA6 downregulation on smooth muscle marker expression, GapmeRs were used to downregulate GATA6. GapmeR-mediated knockdown of GATA6 reduced *ACTA2* and *CNN1* gene expression (Figure 1C). No significant downregulation of *SYNPO2* was observed (Figure 1C).

### GATA6 induces migration of human coronary artery smooth muscle cells

**FIGURE 2:**
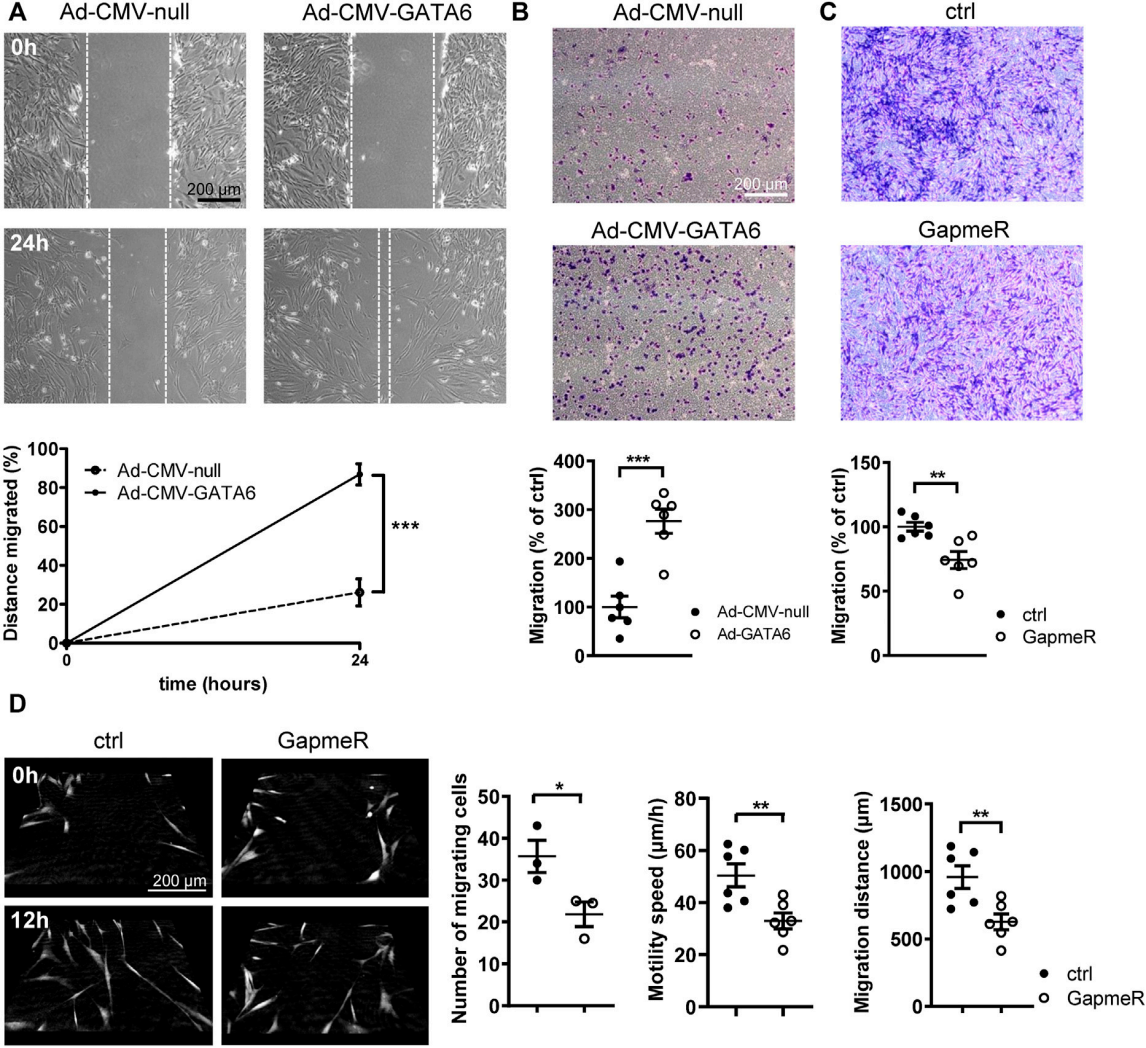
GATA6 promotes migration of human vascular smooth muscle cells. Human coronary artery smooth muscle cells were transduced with Ad-CMV-GATA6/Ad-CMV-null or transfected with GapmeRs against GATA6. Cell migration was assessed using a **(A)** wound-healing assay, **(B,C)** transwell migration assay (24 h). **(D)** Digital holographic cytometry was used to measure migration of human coronary artery smooth muscle cells after GapmerRs transfection to downregulate GATA6. Photos were taken at the indicated time points. Number of migrating cells were analyzed after 24 h. Migration speed and distance after 19 h. Data are presented as mean ± SEM (*n* = 3–6). *p < 0.05, **p < 0.01, ***p < 0.001.

Phenotypically modified SMCs are a hallmark of many vascular diseases, characterized by increased proliferative and migratory rates. It is well established that GATA6 facilitates anti-proliferative effects on VSMCs, preventing neointimal formation following vascular injury (Mano et al., 1999). However, the effect of GATA6 on VSMC migration is not well documented and remains poorly understood. A wound healing assay was initially used to determine the effect of GATA6 overexpression on cell migration. Surprisingly, adenoviral overexpression of GATA6 dramatically enhanced migration of HCASMCs (Figure 2A). This finding was further corroborated using a transwell migration assay, obtaining similar results (Figure 2B). Consistent with these findings, loss of function studies using GapmeRs against GATA6 resulted in reduced cellular motility following downregulation of GATA6 (Figure 2C). This was further supported by live imaging and tracking of individual cell movement over time using digital holographic cytometry (Figure 2D). Both migration speed and distance were significantly reduced in cells treated with GATA6 GapmeRs. The live imaging also confirmed that wound healing in this assay is almost exclusively due to cell migration, and not cell proliferation. All together, these data suggest a role for GATA6 in regulating migration of HCASMCs.

### Transcriptome profiling reveals multiple changes associated with GATA6 overexpression in VSMCs

**FIGURE 3:**
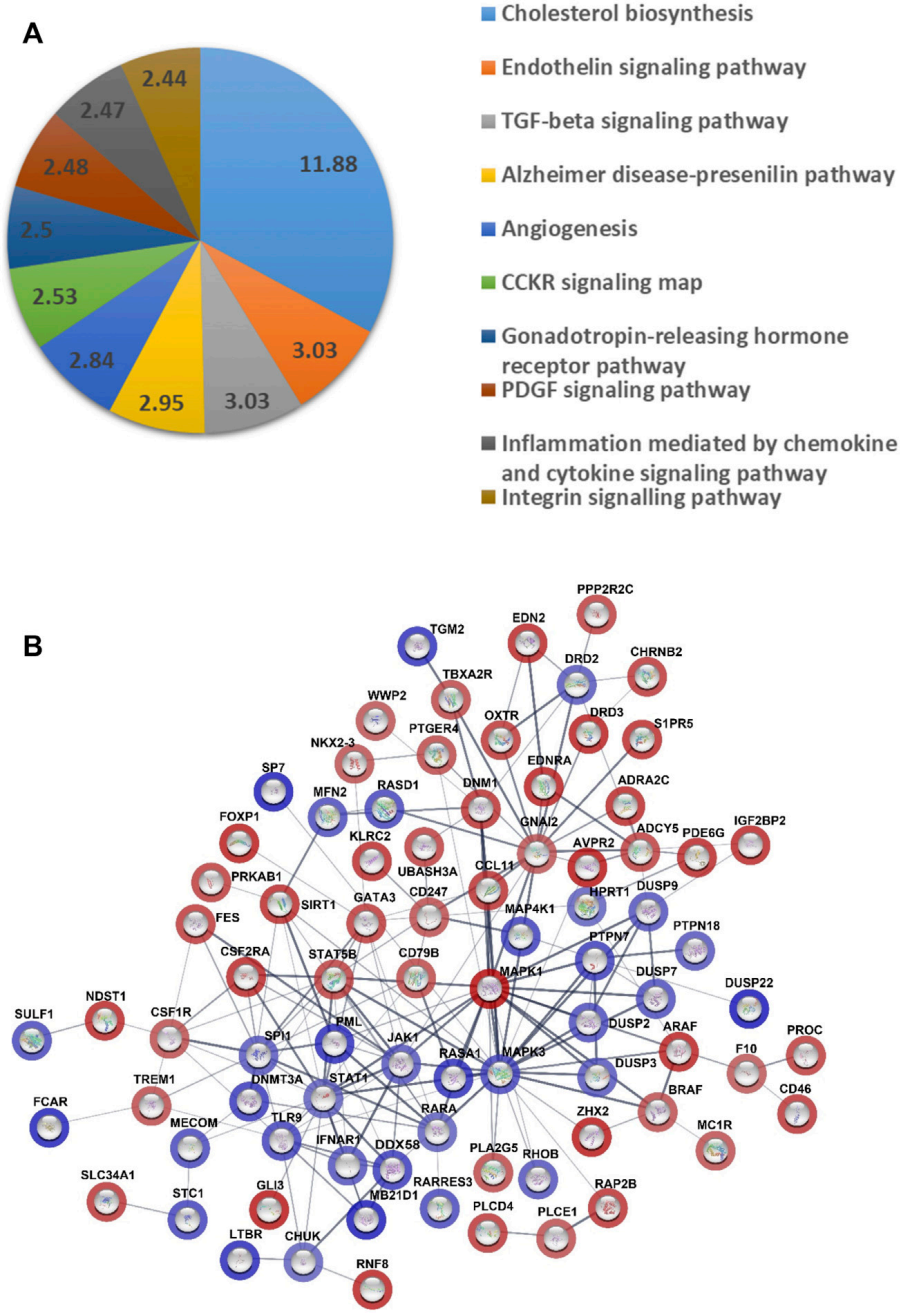
Analysis of gene expression data from microarray of the cells overexpressing GATA6 and control. **(A)** Pie chart of pathway gene analysis of differentially expressed genes. Analysis was done using PANTHER (17.0 Release). The numbers represent fold enrichment. Only results with false discovery rate < 0.05 are presented. **(B)** Prediction of master regulators was done by QIAGEN Ingenuity Pathway Analysis software. A Network of master regulator was done by STRING database (version 11.5). The color shades represent the degree of activation/inhibition ranging from dark blue (most active) to dark red (most inhibited). The lines indicate the strength of data support such interaction. 17 master regulators were excluded from the network, since there was no evidence supporting any interaction with the other master regulators.

To further investigate the molecular basis for the action of GATA6 we performed a microarray on cells transduced with either Ad-CMV-GATA6 or vehicle (Ad-CMV-null) to identify genes regulated by GATA6. This analysis revealed 739 upregulated and 248 downregulated genes with *q* = 0. The top 50 most up- and downregulated genes are listed in (Supplementary Table S1). Pathway enrichment analysis on the microarray data revealed contribution of pathways that are possibly involved in cell migration, including endothelin and TGF-β signaling pathway (3.03, <0.05), angiogenesis (2.84, <0.05), PDGF signaling pathway (2.48, <0.05), inflammation mediated by chemokines (2.47, <0.05), and integrin signaling pathway (2.44, <0.05) (Figure 3A).

Depending on the fold changes of the genes in the microarray, a prediction of upstream master regulators was done by Ingenuity Pathway Analysis software (Qiagen) and these master regulators were given a Z-score to reflect their degree of activation or inhibition. The top 100 master regulators are presented as a network in (Figure 3B) and their fold changes and Z-score in (Supplementary Table S2). Among these master regulators there were proteins that reflect an activation state of mitogen activated protein kinase (MAPK), MAP4K1 (Z-score = 4.15), MAPK3 (3.95), RASA1 (4.67), and STAT1 (3.13). TGFB2/3, SMAD1/3, and BMP2/4 alternatively contributed to prediction of 82 out of 100 master regulators and they were all present in prediction of 18 master regulators, such as RASA1, MAP4K1, RHOB (3.57). However, there were three of dual-specificity MAP kinase phosphatases among the activated master regulators (DUSP2, DUSP7, and DUSP9) which act as negative regulators for MAPK kinases.

**FIGURE 4:**
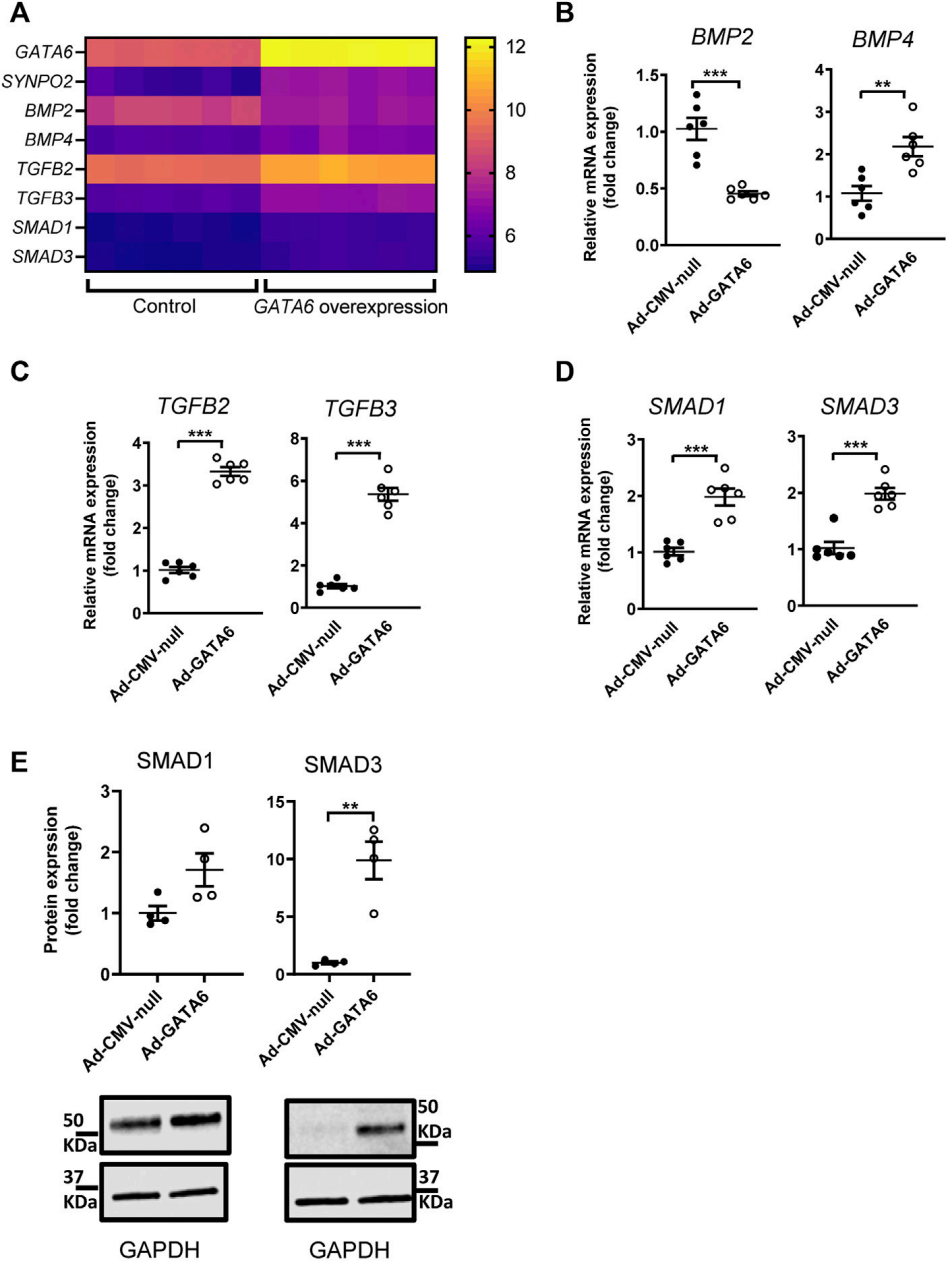
Identification of genes regulated by GATA6 expression. A complete microarray screen of GATA6-sensitive gene transcription was performed on human coronary artery smooth muscle cells transduced with 100 MOI Ad-CMV-GATA6 or Ad-CMV-null (vehicle) as a control. Expression values for selected genes from the microarray from each sample (*n* = 6) **(A)**. Validation of selected genes by qRT-PCR **(B–D)** and western blot **(E)**. Data are presented as mean ± SEM (*n* = 6). **p < 0.01, ***p < 0.001.

An analysis of up- and down-regulation of specific genes in the enriched pathways revealed that TGF-beta signaling was the most likely potential mediator of GATA6 induced cell migration specific genes. Several members of TGF-β superfamily and downstream signaling pathways, including bone morphogenetic proteins (BMPs), TGF-βs and SMADS were upregulated in GATA6-overexpressiong cells (Figure 4A). Validation of the observed changes for these selected targets was done by qRT-PCR (Figures 4B–D) and western blotting (Figure 4E). Collectively, these data showed that members of TGF-β and MAPK signaling pathways are among the affected genes after GATA6 overexpression (Supplementary Tables S3, S4). Further studies are needed to elucidate their role in GATA6-mediated cell migration.

GATA6 was recently shown to regulate myocardin expression in visceral smooth muscle, and myocardin is known to interact with GATA6 to control smooth muscle specific gene expression (Oh et al., 2004; Kurz et al., 2022). However, the microarray analysis did not suggest significant change in myocardin expression following GATA6-overexpression (FC: 0.69; q: 0.28).

## Discussion

Despite great advances in medical therapy with techniques including balloon angioplasty and stenting, cardiovascular diseases remain the leading cause of death worldwide. A large body of evidence demonstrates that the evolutionary conserved transcription factor GATA6 protects against injury-induced vascular lesions by modulating SMC plasticity. Although a role for GATA6 in phenotypic modulation has been proposed, the underlying mechanisms whereby GATA6 modulates this process is not well-understood. Several studies have demonstrated a positive effect of GATA6 on SMC differentiation. In agreement with this, our results confirmed a significant induction of SMC markers following upregulation of GATA6 in human coronary smooth muscle cells. Furthermore, silencing of GATA6 downregulated the expression of *ACTA2* and *CNN1*.

It is well known that SMC proliferation and migration play a pivotal role for vascular disease development and progression. While several studies have demonstrated a major role for GATA6 in cell cycle arrest, less is known about its role in regulating cell motility. Dysregulation of GATA6 has been connected to cell migration in non-smooth muscle cells including cancer cells (Sun and Yan, 2020). In accordance with our results, cell migration of established colorectal cancer cell (CRC) lines is decreased upon GATA6 knockdown and enhanced by GATA6 overexpression (Shen et al., 2013). The mechanism whereby GATA6 mediates CRC migration is not fully understood but BMP4 has been suggested to play a role in this process (Shen et al., 2013). This is consistent with our study demonstrating a >2-fold increase of BMP4 mRNA expression in GATA6-overexpressing cells. However, GATA6 has been reported to both promote and inhibit cell migration in other types of cancer including lung cancer, suggesting a complex role of GATA6 for this effect (reviewed in Sun and Yan, 2020).

To our knowledge, only one earlier study has demonstrated a link between GATA6 and smooth muscle migration, which suggests that GATA6 knockdown leads to an increased VSMC migration (Zhuang et al., 2019). This notion, however, is in contrast with our findings which demonstrate that GATA6 knockdown inhibits VSMC migration. Notably, the results in our study were verified using three different experimental methods to study cell migration. In addition, both overexpression and loss-of-function approaches were employed to assess the role of GATA6 in SMCs. The discrepancy between our study and Zhuang et al. (Zhuang et al., 2019). may be due to the experimental approach used or the heterogeneity of VSMCs (Majesky, 2007). For example, the study by Zhuang et al. used aortic smooth muscle cells whereas our study was performed on coronary artery smooth muscle cells, which may have different properties. Thus, the results of our study may be more relevant for vascular disease in coronary arteries.

Our results support a complex role of GATA6 in the regulation of smooth muscle phenotype, involving both upregulation of specific contractile smooth muscle markers and increased smooth muscle cell migration. Generally, differentiated SMCs are considered quiescent, non-proliferative and non-migratory, while dedifferentiated cells downregulate contractile proteins, upregulate extracellular matrix (ECM) synthesis, are migratory and proliferative. Hence, cell differentiation and migration are frequently considered mutually exclusive events, which may be a misconception. Earlier studies have demonstrated that insulin maintains a differentiated state of smooth muscle *via* phosphoinositide 3-kinase (PI3K) pathway while promoting cell migration *via* (MAPK) signaling pathway, which was predicted to be activated in our microarray dataset (Wang et al., 2003).

Cell migration is dependent on changes in the structure of the cytoskeleton, a process driven by dynamic treadmilling of actin filaments. A large number of actin-binding proteins coordinate the assembly of actin into various structures including stress fibers, actin networks and actin bundles. These structural changes form membrane protrusions including lamellipodia and filopodia at the leading edge of the cell acting as sensors to direct locomotion. In addition to actin remodeling, cell migration requires myosin II to generate force *via* actomyosin networks for the retraction of the rear and to propel the cell forward (Gerthoffer, 2007). Studies have demonstrated that proteins involved in regulating the actin cytoskeleton are upregulated in several cancers promoting cell migration and an invasive and malignant phenotype (Yamaguchi and Condeelis, 2007). Hence, the upregulation of cytoskeletal and contractile genes observed following GATA6 overexpression may play an important role in regulating cellular motility. Altogether, it is possible that GATA6 plays a multifaceted role, similar to that of myocardin related transcription factors (MRTF) and YAP/TAZ, which have been demonstrated to regulate SMC-specific gene expression yet are ubiquitously expressed and increased in vascular injury and disease (Wang et al., 2002; Minami et al., 2012; Wang et al., 2012; Ito et al., 2020; Zhu et al., 2020; Daoud et al., 2021; Gao et al., 2021; Daoud et al., 2022).

We identified several GATA6-regulated genes belonging to the TGF-β superfamily as part of microarray dataset analyses. Dysregulation of TGF-β superfamily signaling pathways is associated with several human disease states including atherosclerosis (Grainger et al., 1995; Mallat et al., 2001; Cipollone et al., 2004), hypertension (Zacchigna et al., 2006) and restenosis (Majesky et al., 1991; Nikol et al., 1992; Ward et al., 1997; Mallawaarachchi et al., 2005; Xie et al., 2019). While TGF-β is thought to protect against atherosclerosis, accumulating evidence indicate a positive role for TGF-β signaling in the development of hypertension and restenosis. In parallel, increased VSMC migration is considered to play an important role in stabilizing atherosclerotic plaques whereas in hypertension and restenosis, VMSC migration is involved in disease progression through neointima formation. The TGF-β superfamily has a diverse array of effects on SMCs, many of which are relevant for the development and progression of neointima formation such as cell proliferation, migration and matrix protein synthesis (Rasmussen et al., 1995; Ryer et al., 2006; Xie et al., 2019). The role of TGF-β superfamily on SMC proliferation and migration is controversial (Goodman and Majack, 1989; Agrotis et al., 1994) as some studies have demonstrated a positive effect (Koyama et al., 1990; Willette et al., 1999; Tsai et al., 2009), whereas others support an inhibitory effect (Morisaki et al., 1991; Mii et al., 1993; Kim et al., 2015). In the current study, matrix metalloproteases, integrins and chemokines, all known to regulate cell migration, were differentially expressed following GATA6 overexpression. This suggests that the changes in mRNA expression observed in the microarray, are consistent with the functional effects observed following increased GATA6 expression. However, further studies are required to clarify the precise mechanism underlying increased GATA6-induced migration.

The expression levels of GATA6 in cultured cells is strongly induced by transduction of GATA6 adenovirus and potentially higher compared to the *in vivo* situation. However, GATA6 has been reported to be highly expressed *in vivo* (Yin and Herring, 2005), whereas the expression level in cultured SMC was relatively low. Future studies will need to determine if GATA6-inhibition can prevent SMC migration in vascular disease.

In conclusion, in this study we provide further insight into the functional role of GATA6 in vascular smooth muscle. We demonstrate that GATA6 promotes migration of HCASMCs, which is of clinical relevance as VSMC migration is a key step in the development and progression of vascular diseases. Finding ways to reduce GATA6-mediated migration may potentiate the positive effects of GATA6. For future work it will be important to elucidate the involvement of TGF-β superfamily in GATA6-induced cell migration and to determine if GATA6 or members of the TGF-β superfamily could be used to promote atherosclerotic plaque stability by stimulating SMCs to strengthen the fibrotic cap. A better understanding of the relationship between GATA6 and cell migration could provide a deeper insight into the molecular mechanisms through which GATA6 regulates smooth muscle phenotype and enable us to identify therapeutic strategies in vascular disease.

## Data Availability

The datasets presented in this study can be found in online repositories. The names of the repository/repositories and accession number(s) can be found below: https://www.ncbi.nlm.nih.gov/geo/, GSE216686.
