# Supplementary material for: Transcription factor GATA6 promotes migration of human coronary artery smooth muscle cells in vitro
Source: Front Physiol. 2022 Nov 29;13:1054819. doi: 10.3389/fphys.2022.1054819 (PMC9744938; doi:10.3389/fphys.2022.1054819)
Supplement: Supplementary file 1 [file DataSheet1.PDF]

Supplemental Material

Supplemental Table 1. The top 50 upregulated and top 50 downregulated genes (q=0) following GATA6 overexpression. Gene expression analysis was performed using Affimatrix human gene array on cultured smooth muscle cells transduced with 100MOI Ad-CMV-GATA6 for 144 hours.

Gene symbol	Gene description	Fold change
**FDCSP**	follicular dendritic cell secreted protein	9.36
**BST2**	bone marrow stromal cell antigen 2	8.69
**GATA6**	GATA binding protein 6	8.39
**IFIT1**	interferon-induced protein with tetratricopeptide repeats 1	8.09
**MX1**	myxovirus (influenza virus) resistance 1. interferon-inducible protein p78 (mouse)	7.54
**CD14**	CD14 molecule	7.16
**CALB2**	calbindin 2	6.62
**OAS1**	2'-5'-oligoadenylate synthetase 1. 40/46kDa	6.58
**IFI6**	interferon. alpha-inducible protein 6	6.44
**IFI44L**	interferon-induced protein 44-like	6.42
**HERC6**	HECT and RLD domain containing E3 ubiquitin protein ligase family member 6	6.23
**HIST1H3H**	histone cluster 1. H3h	6.13
**CFI**	complement factor I	5.92
**SLC14A1**	solute carrier family 14 (urea transporter). member 1 (Kidd blood group)	5.90
**OAS2**	2'-5'-oligoadenylate synthetase 2. 69/71kDa	5.83
**GBP4**	guanylate binding protein 4	5.57
**IFI44**	interferon-induced protein 44	5.33
**MMP12**	matrix metallopeptidase 12 (macrophage elastase)	4.83
**IFIT3**	interferon-induced protein with tetratricopeptide repeats 3	4.79
**SAMHD1**	SAM domain and HD domain 1	4.52
**TMEM150C**	transmembrane protein 150C	4.37
**KEL**	Kell blood group. metallo-endopeptidase	4.34
**HCP5**	HLA complex P5 (non-protein coding)	4.25
**ACE2**	angiotensin I converting enzyme 2	4.21
**SEBOX // VTN**	SEBOX homeobox // vitronectin	4.20
**RPLP0P2**	ribosomal protein. large. P0 pseudogene 2	4.15
**MX2**	myxovirus (influenza virus) resistance 2 (mouse)	4.06
**SLPI**	secretory leukocyte peptidase inhibitor	4.04
**IFITM1**	interferon induced transmembrane protein 1	3.96
**DOCK11**	dedicator of cytokinesis 11	3.95
**ENPP3**	ectonucleotide pyrophosphatase/phosphodiesterase 3	3.89
**SERPINB9**	serpin peptidase inhibitor. clade B (ovalbumin). member 9	3.81
**TAC3**	tachykinin 3	3.79
**MMD**	monocyte to macrophage differentiation-associated	3.78

**GPRC5B**	G protein-coupled receptor. family C. group 5. member B	3.71
**OLR1**	oxidized low density lipoprotein (lectin-like) receptor 1	3.67
**CEACAM1**	carcinoembryonic antigen-related cell adhesion molecule 1 (biliary glycoprotein)	3.54
**CYP2S1**	cytochrome P450. family 2. subfamily S. polypeptide 1	3.50
**PKP2**	plakophilin 2	3.44
**SERPINB1**	serpin peptidase inhibitor. clade B (ovalbumin). member 1	3.37
**HIST1H2AI**	histone cluster 1. H2ai	3.32
**CROT**	carnitine O-octanoyltransferase	3.32
**CPA4**	carboxypeptidase A4	3.28
**CLU**	clusterin	3.28
**OAS3**	2'-5'-oligoadenylate synthetase 3. 100kDa	3.27
**AQP11**	aquaporin 11	3.26
**EGFL6**	EGF-like-domain. multiple 6	3.24
**HCP5**	HLA complex P5 (non-protein coding)	3.18
**ITGA6**	integrin. alpha 6	3.15
**SYNPO2**	synaptopodin 2	3.15

Gene symbol	Gene description	Fold change
**A2M**	alpha-2-macroglobulin	0.15
**IL24**	interleukin 24	0.22
**SCG5**	secretogranin V (7B2 protein)	0.22
**CCL11**	chemokine (C-C motif) ligand 11	0.23
**RGCC**	regulator of cell cycle	0.25
**PLCL1**	phospholipase C-like 1	0.27
**PALMD**	palmdelphin	0.28
**IBSP**	integrin-binding sialoprotein	0.28
**NEFM**	neurofilament. medium polypeptide	0.28
**LRRC38**	leucine rich repeat containing 38	0.28
**IL11**	interleukin 11	0.29
**SPANXN4**	SPANX family. member N4	0.29
**KRTAP7-1**	keratin associated protein 7-1 (gene/pseudogene)	0.31
**LOC541472**	uncharacterized LOC541472	0.31
**LOC100292922**	ankyrin repeat domain-containing protein 30B-like	0.32
**KRTAP2-3**	keratin associated protein 2-3	0.32
**MRGPRX3**	MAS-related GPR. member X3	0.33
**LMCD1**	LIM and cysteine-rich domains 1	0.33
**LINC00862**	long intergenic non-protein coding RNA 862	0.33
**LOXL4**	lysyl oxidase-like 4	0.34
**ADAM12**	ADAM metallopeptidase domain 12	0.35
**OLAH**	oleoyl-ACP hydrolase	0.37
**GDF6**	growth differentiation factor 6	0.38
**TREM1**	triggering receptor expressed on myeloid cells 1	0.38

**TNFRSF10D**	tumor necrosis factor receptor superfamily. member 10d. decoy with truncated death domain	0.38
**C15orf54**	chromosome 15 open reading frame 54	0.39
**EDN1**	endothelin 1	0.39
**ANGPT2**	angiopoietin 2	0.40
**TRIM55**	tripartite motif containing 55	0.40
**MME-AS1**	MME antisense RNA 1	0.40
**GABBR2**	gamma-aminobutyric acid (GABA) B receptor. 2	0.41
**IL6**	interleukin 6 (interferon. beta 2)	0.41
**DAW1**	dynein assembly factor with WDR repeat domains 1	0.41
**PLAU**	plasminogen activator. urokinase	0.41
**LOC100506394**	uncharacterized LOC100506394	0.42
**LCP1**	lymphocyte cytosolic protein 1 (L-plastin)	0.42
**RPSAP52**	ribosomal protein SA pseudogene 52	0.42
**FIBIN**	fin bud initiation factor homolog (zebrafish)	0.42
**LOC646999**	akirin 1 pseudogene	0.42
**VIPR1**	vasoactive intestinal peptide receptor 1	0.43
**CENPN**	centromere protein N	0.43
**ARSJ**	arylsulfatase family. member J	0.44
**XYLT1**	xylosyltransferase I	0.44
**GREM1**	gremlin 1. DAN family BMP antagonist	0.44
**VGLL3**	vestigial like 3 (Drosophila)	0.44
**LINC00312**	long intergenic non-protein coding RNA 312	0.45
**MYLK**	myosin light chain kinase	0.45
**MAMDC2**	MAM domain containing 2	0.45
**CCL8**	chemokine (C-C motif) ligand 8	0.45
**TNC**	tenascin C	0.45

Supplemental Table 2. The top 100 master regulators of gene expression dataset following GATA6 overexpression. The Master regulators were sorted based on absolute Z-score. The positive Z-score indicates activation, whereas the negative Z-score indicates inhibition.

Master Regulator	Molecule Type	Fold Change	Z-score	Absolute Z-score
**TNK1**	kinase	-1.01	5.74	5.74
**DUSP22**	phosphatase	-1.01	5.35	5.35
**MAPK1**	kinase	1.07	-5.25	5.25
**SP7**	transcription regulator	-1.11	5.24	5.24
**CGAS**	enzyme	1.30	5.19	5.19
**ANO6**	ion channel	1.09	5.09	5.09
**SCLT1**	transporter	-1.23	5.09	5.09
**FCAR**	transmembrane receptor	-1.01	5.05	5.05
**AVPR2**	G-protein coupled receptor	1.11	-5.01	5.01
**GLI3**	transcription regulator	1.18	-4.90	4.90

**ZHX2**	transcription regulator	1.03	-4.87	4.87
**LTBR**	transmembrane receptor	1.10	4.84	4.84
**FOXP1**	transcription regulator	-1.10	-4.80	4.80
**PML**	transcription regulator	1.43	4.75	4.75
**ELF4**	transcription regulator	-1.03	4.75	4.75
**DRD3**	G-protein coupled receptor	-1.04	-4.71	4.71
**EDNRA**	transmembrane receptor	1.45	-4.67	4.67
**RASA1**	transporter	-1.19	4.67	4.67
**RNF8**	enzyme	1.22	-4.64	4.64
**ARAF**	kinase	-1.02	-4.64	4.64
**TGM2**	enzyme	-2.13	4.63	4.63
**CSF2RA**	transmembrane receptor	-1.04	-4.52	4.52
**EPS8**	peptidase	-1.16	-4.52	4.52
**NDST1**	enzyme	-1.19	-4.52	4.52
**EDN2**	growth factor	-1.05	-4.48	4.48
**IGF2BP2**	translation regulator	-1.14	-4.48	4.48
**PDE6G**	enzyme	-1.09	-4.48	4.48
**ADRA2C**	G-protein coupled receptor	-1.17	-4.45	4.45
**SIRT1**	transcription regulator	-1.05	-4.42	4.42
**DNMT3A**	enzyme	1.11	4.39	4.39
**KLRC2**	transmembrane receptor	-1.10	-4.37	4.37
**S1PR5**	G-protein coupled receptor	-1.12	-4.36	4.36
**CMKLR1**	G-protein coupled receptor	-1.00	-4.35	4.35
**DDX58**	enzyme	1.72	4.31	4.31
**PTPN7**	phosphatase	1.03	4.23	4.23
**RAP2B**	enzyme	1.27	-4.22	4.22
**CCL11**	cytokine	-4.39	-4.20	4.20
**DNM1**	enzyme	1.01	-4.20	4.20
**MAP4K1**	kinase	1.07	4.15	4.15
**CTH**	enzyme	1.28	4.14	4.14
**CD46**	transmembrane receptor	1.15	-4.13	4.13
**SLC34A1**	transporter	-1.02	-4.12	4.12
**TLR9**	transmembrane receptor	1.13	4.10	4.10
**STC1**	kinase	-1.20	4.09	4.09
**OXTR**	G-protein coupled receptor	1.34	-4.08	4.08
**CHRNB2**	transmembrane receptor	-1.02	-4.08	4.08
**PLCD4**	enzyme	1.19	-4.08	4.08
**NRP2**	kinase	-1.44	4.05	4.05
**FES**	kinase	1.08	-4.03	4.03
**GATA3**	transcription regulator	1.02	-4.02	4.02
**RASD1**	enzyme	-1.15	4.00	4.00

**MAPK3**	kinase	-1.08	3.95	3.95
**PROC**	peptidase	1.05	-3.95	3.95
**GRM4**	G-protein coupled receptor	-1.09	-3.94	3.94
**PRKAB1**	kinase	1.16	-3.94	3.94
**MECOM**	transcription regulator	-1.12	3.92	3.92
**PLAAT4**	enzyme	1.91	3.92	3.92
**GLRX**	enzyme	-1.18	-3.91	3.91
**SULF1**	enzyme	1.26	3.90	3.90
**PPP2R2C**	phosphatase	-1.15	-3.88	3.88
**UBASH3A**	enzyme	-1.06	-3.88	3.88
**ADCY5**	enzyme	1.01	-3.88	3.88
**STAT5B**	transcription regulator	1.19	-3.81	3.81
**MFN2**	enzyme	1.00	3.79	3.79
**DUSP2**	phosphatase	-1.12	3.76	3.76
**DUSP7**	phosphatase	1.06	3.72	3.72
**ELF1**	transcription regulator	1.01	-3.71	3.71
**NKX2-3**	transcription regulator	1.01	-3.71	3.71
**ATE1**	enzyme	1.08	-3.68	3.68
**MGAT5**	enzyme	1.74	-3.67	3.67
**TBXA2R**	G-protein coupled receptor	1.03	-3.62	3.62
**CHKA**	kinase	1.21	-3.61	3.61
**CDK5R1**	kinase	1.00	3.59	3.59
**CD79B**	transmembrane receptor	-1.06	-3.59	3.59
**PLA2G5**	enzyme	1.21	-3.59	3.59
**RHOB**	enzyme	-1.45	3.57	3.57
**DUSP9**	phosphatase	-1.09	3.57	3.57
**IFNAR1**	transmembrane receptor	1.10	3.55	3.55
**PTGER4**	G-protein coupled receptor	1.03	-3.55	3.55
**TREM1**	transmembrane receptor	-2.65	-3.50	3.50
**PTPN18**	phosphatase	-1.03	3.49	3.49
**DUSP3**	phosphatase	-1.11	3.48	3.48
**MC1R**	G-protein coupled receptor	1.27	-3.45	3.45
**PLCE1**	enzyme	-1.04	-3.44	3.44
**DRD2**	G-protein coupled receptor	1.67	3.44	3.44
**CD247**	transmembrane receptor	-1.06	-3.43	3.43
**JAK1**	kinase	1.03	3.43	3.43
**BRAF**	kinase	1.05	-3.41	3.41
**CSF1R**	kinase	1.13	-3.40	3.40
**SPI1**	transcription regulator	-1.10	3.40	3.40
**APOC1**	transporter	1.03	3.33	3.33
**POR**	enzyme	1.15	3.30	3.30

**WWP2**	enzyme	1.24	-3.27	3.27
**GNAI2**	enzyme	-1.06	-3.22	3.22
**RARA**	ligand-dependent nuclear receptor	1.05	3.19	3.19
**HPRT1**	enzyme	-1.19	3.19	3.19
**F10**	peptidase	1.01	-3.16	3.16
**STAT1**	transcription regulator	2.08	3.13	3.13
**CHUK**	kinase	-1.06	3.12	3.12
**MMP17**	peptidase	1.02	-3.10	3.10

Supplemental Table 3. The overlapped genes between the significant genes from the array and TGFB signaling pathway. Genes with (q=0) from Affimatrix human gene array were overlapped with genes in TGFB signaling pathway using KEGG PATHWAY list (hsa04350).

Gene symbol	Gene description	Fold change
**GDF6**	growth differentiation factor 6	0.38
**BMP2**	bone morphogenetic protein 2	0.46
**INHBA**	inhibin, beta A	0.51
**NOG**	noggin	0.59
**SMAD1**	SMAD family member 1	1.45
**SMAD3**	SMAD family member 3	1.50
**BMPR2**	bone morphogenetic protein receptor, type II (serine/threonine kinase)	1.64
**ID4**	inhibitor of DNA binding 4, dominant negative helix-loop-helix protein	1.74
**BMP4**	bone morphogenetic protein 4	1.83
**DCN**	decorin	1.91
**TGFB3**	transforming growth factor, beta 3	2.31
**TGFB2**	transforming growth factor, beta 2	2.34

Supplemental Table 4. The overlapped genes between the significant genes from the array and MAPK signaling pathway. Genes with (q=0) from Affimatrix human gene array were overlapped with genes in MAPK signaling pathway using KEGG PATHWAY list (hsa04010).

Gene symbol	Gene description	Fold change
**FGF7**	fibroblast growth factor 7	0.47
**DUSP1**	dual specificity phosphatase 1	0.49
**DUSP5**	dual specificity phosphatase 5	0.60
**FGF1**	fibroblast growth factor 1 (acidic)	0.62
**GADD45B**	growth arrest and DNA-damage-inducible, beta	0.62
**NR4A1**	nuclear receptor subfamily 4, group A, member 1	0.63
**MKNK2**	MAP kinase interacting serine/threonine kinase 2	1.47
**MEF2C**	myocyte enhancer factor 2C	1.49
**RPS6KA5**	ribosomal protein S6 kinase, 90kDa, polypeptide 5	1.50

**DUSP6**	dual specificity phosphatase 6	1.52
**MAP3K8**	mitogen-activated protein kinase kinase kinase 8	1.52
**PLA2G12A**	phospholipase A2, group XIIA	1.54
**ARRB2**	arrestin, beta 2	1.60
**PPM1B**	protein phosphatase, Mg2+/Mn2+ dependent, 1B	1.63
**RASGRF2**	Ras protein-specific guanine nucleotide-releasing factor 2	1.64
**IL1R1**	interleukin 1 receptor, type I	1.83
**DUSP4**	dual specificity phosphatase 4	1.87
**TGFB3**	transforming growth factor, beta 3	2.31
**TGFB2**	transforming growth factor, beta 2	2.34
**CD14**	CD14 molecule	7.16
